# Patient survival and kidney transplantation in different dialysis modalities under PD First Policy Thailand

**DOI:** 10.1371/journal.pone.0336954

**Published:** 2025-11-19

**Authors:** Pornpen Sangthawan, Thammasin Ingviya, Songyos Rajborirug, Jirayut Janma, Siribha Changsirikulchai

**Affiliations:** 1 Division of Nephrology, Department of Medicine, Faculty of Medicine, Prince of Songkla University, Songkhla, Thailand; 2 Department of Clinical Research and Medical Data Science, Faculty of Medicine, Prince of Songkla University, Songkhla, Thailand; 3 Department of Epidemiology, Faculty of Medicine, Prince of Songkla University, Songkhla, Thailand; 4 Division of Nephrology, Department of Medicine, Faculty of Medicine, Srinakharinwirot University, Nakhonnayok, Thailand; University of Toronto Temerty Faculty of Medicine, CANADA

## Abstract

**Background:**

Thailand implemented a peritoneal dialysis (PD)-first policy under its universal health coverage (UHC) from 2008 to 2022. This study aims to describe patient survival during dialysis and after kidney transplantation (KT), and to identify factors associated with survival in these periods among UHC-covered patients undergoing PD, hemodialysis (HD), or transitioning between dialysis modalities.

**Methods:**

This retrospective study analyzed data from patients receiving PD, HD, or KT, recorded by the National Health Security Office (NHSO) between January 2013 and December 2021. Patients were categorized into four groups: PD, HD, PD-to-HD transition, and HD-to- PD transition. Survival factors were analyzed using Cox proportional hazards models.

**Results:**

Among 81,572 patients receiving kidney replacement therapy, 38.9% were on PD, 35.3% were on HD, 10.2% transitioned from PD to HD, and 15.6% transitioned from HD to PD. Patients transitioning from PD to HD had superior 3- and 5-year survival rates compared to the other three groups. Survival outcomes were significantly influenced by age at dialysis initiation, diabetes, and comorbidities. Overall, 1,517 patients (1.9%) received KT: 70.4% had PD, 19.8% HD, and 9.8% had transitioned. Median follow-up time before KT was 94.5 months. Post-KT survival rates were comparable across dialysis groups. Factors associated with post-KT survival were age, cardiac disease, antibody-mediated rejection, and delayed graft function.

**Conclusions:**

Under Thailand’s PD-first policy, starting with PD and later switching to HD was linked to better survival than staying on a single modality or switching from HD to PD. A higher proportion of PD patients underwent KT compared to HD patients. Post-KT survival rates remained similar across all dialysis modalities. These findings underscore the importance of individualized dialysis modality selection and proactive transition planning to optimize patient outcomes.

## Introduction

End-stage kidney disease (ESKD) represents a significant and growing global health challenge, driven by aging populations and the increasing prevalence of diabetes and hypertension [1,2]. Kidney replacement therapy (KRT) plays a critical role in managing ESKD, with peritoneal dialysis (PD), hemodialysis (HD), and kidney transplantation (KT) being the primary treatment options. While KT offers the most favorable long-term outcomes, dialysis remains the predominant modality for the majority of patients worldwide due to limitations in donor availability and accessibility [3]. Thailand implemented the PD-first policy in 2008 as part of its Universal Health Coverage (UHC) scheme to address inequities in dialysis access and ensure equitable treatment for ESKD patients. This policy designated PD as the initial dialysis modality for all UHC-covered ESKD patients, except for those with specific medical contraindications. By prioritizing PD, the policy aimed to mitigate key challenges such as economic constraints, geographic barriers, and disparities in healthcare access. The UHC scheme itself covers the majority of Thai citizens, defined as nationals with a valid 13-digit national identification number who are not eligible for other government health insurance funds. Although the PD-first policy has successfully expanded dialysis coverage and improved equity, questions remain about its long-term implications, particularly regarding patient survival. Several studies have compared patient survival and KT outcomes between PD and HD [4–7]. The results varied due to the differences in criteria, statistical methodology, and length of follow-up. The objective of this study is to describe an overview of patient characteristics and survival patterns during dialysis and after KT, as well as to identify factors associated with survival among UHC-covered patients undergoing PD, HD, or transitioning between modalities, without testing a specific hypothesis, within the context of Thailand’s PD-first policy. The findings may inform countries considering the adoption of PD-first policies or implementing KRT for ESKD patients.

## Methods

### Study population

This retrospective cohort study included incident dialysis patients aged 18 years or older who were covered by the UHC program and survived for at least 60 days after dialysis initiation. The study period spanned from January 2013 to December 2021, during which Thailand’s PD-first policy was in effect. Data were accessed for research purposes in 1^st^ November, 2022. Under this policy, patients with ESKD were expected to initiate PD unless they had contraindications making PD unsuitable, in which case they could begin with HD. Data were obtained from the National Health Security Office (NHSO) database, which includes inpatient claims, outpatient records, kidney replacement therapy (KRT) data, and Data Management Information System. Based on their dialysis modality, patients were categorized into four groups: PD, HD, PD-to-HD transition, and HD-to-PD transition. Modality transition was defined as a change in dialysis modality lasting at least 60 consecutive days. This cutoff was chosen to exclude transient transition caused by short-term complications and was comparable to time thresholds used by the USRDS [8]. Patients with more than one transition were excluded because this heterogeneous group was more likely to experience frequent complications, such as peritonitis or access-related infections, resulting in multiple short-duration switches that did not meet this threshold for meaningful modality-specific outcome assessment.

For patients who underwent kidney transplantation (KT), three subgroups were defined based on their dialysis modality before transplantation: PD, HD, and a history of dialysis modality transition (PD-to-HD or HD-to-PD). Preemptive kidney transplants (i.e., those performed without prior dialysis) were excluded from the analysis. This study was approved under the exempt category by the Institutional Review Committee for Research in Human Subjects, Faculty of Medicine, Srinakharinwirot University (SWUEC/X/M-067/2565). As a retrospective study, obtaining informed consent from individual patients was not required. All data were fully anonymized prior to analysis, and patient confidentiality was strictly maintained in compliance with ethical guidelines on data protection.

### Data collection and variables

This study utilized available clinical and demographic data, including age at dialysis initiation, sex, comorbidities, and KRT modalities. Comorbidities examined in this study included diabetes, hypertension, atherosclerotic heart disease, heart failure, dysrhythmia, cerebrovascular disease, peripheral vascular disease, other cardiac conditions, chronic obstructive pulmonary disease (COPD), gastrointestinal (GI) bleeding, liver disease, and cancer.

The analysis of dialysis outcomes considered age at dialysis initiation, sex, diabetes status, comorbidities, and dialysis modality. Comorbidities with low frequency were combined into broader categories for analysis: dysrhythmia was grouped under cardiovascular disease, COPD under airway disease, and GI bleeding under liver disease. Information on certain potential confounders, such as socioeconomic status, nutritional status, and early dropout from dialysis, was not recorded in the NHSO dataset. As a result, these factors could not be accounted for in the analysis.

For KT recipients, only the first KT was included in the analysis. Transplant characteristics assessed included donor type (living-related or deceased donor), type and number of HLA mismatches, delayed graft function (DGF), acute cellular rejection, and acute antibody-mediated rejection. Factors considered in the analysis of KT outcomes included age at transplantation, sex, comorbidities, incidence of rejection, occurrence of DGF, and prior dialysis modality.

### Outcomes

For patients who remained on dialysis throughout the study period, the association of dialysis modality with patient survival was analyzed, with KT considered a censoring event.

The likelihood of undergoing KT was compared across dialysis modalities. In addition, KT recipients were analyzed to assess patient survival and factors associated with survival, based on their prior dialysis modality.

### Statistical analysis

Data are presented as mean and standard deviation for normally distributed variables and median and interquartile range for non-normally distributed variables. All categorical data are expressed as frequencies and percentages. The data were compared by Chi-squared test for categorical variables and Wilcoxon rank-sum test for continuous variables. To evaluate the association between dialysis modality and patient survival, a single multivariable Cox proportional hazards model was applied, adjusting for clinically relevant confounders, including age, sex, and comorbidities. Subsequently, a separate multivariable Cox proportional hazards model specifically for KT recipients was created to assess the relationship between dialysis modalities (pre-transplant) and survival post-transplant. The reason for using a different set of covariates in the KT recipient model is due to the inherent differences in the patient characteristics and relevant prognostic factors within this distinct subgroup, such as specific transplant-related variables not applicable to the general dialysis population. Variable selection was based on existing literature and established associations with survival in dialysis and KT recipients [9–12]. To assess potential effect modification of important clinical factors on the association between dialysis modalities and mortality, subgroup analyses were performed by individually stratifying patients by age, diabetes status, and a combined comorbidity variable. The combined comorbidity variable was constructed as a binary variable (yes/no) indicating the presence of at least one of these conditions (age ≥ 60 years, diabetes mellitus, cardiovascular disease, and cerebrovascular disease). Within each stratum, a multivariable Cox proportional hazards model was applied to estimate adjusted HRs across dialysis modalities with specific covariates adjusted. For the stratum age, the model was adjusted for diabetes mellitus, gender, and all other individual comorbid conditions listed (excluding age, which defined the stratum itself). For the stratum diabetes mellitus, the model was adjusted for age, gender, and all other individual comorbid conditions listed (excluding diabetes mellitus, which defined the stratum itself). For the stratum combined comorbidity, the model was adjusted for gender and other individual comorbidities except cardiovascular disease and cerebrovascular disease. The statistical based collinearity, such as variance inflation factor (VIF), was not performed since they might be misled as described by KIM [13]. Thus, the covariates were selected with clinical plausibility in mind to minimize redundancy and maintain model parsimony. Due to selection bias introduced by the PD-first policy, propensity score adjustment or inverse probability weighting could not be applied to mitigate this bias. All statistical analyses were performed using the R program version 3.6.2 (R core team, Austria). A p-value of less than 0.05 was considered statistically significant.

## Results

### Patient characteristics in different dialysis modalities

**Fig 1:**
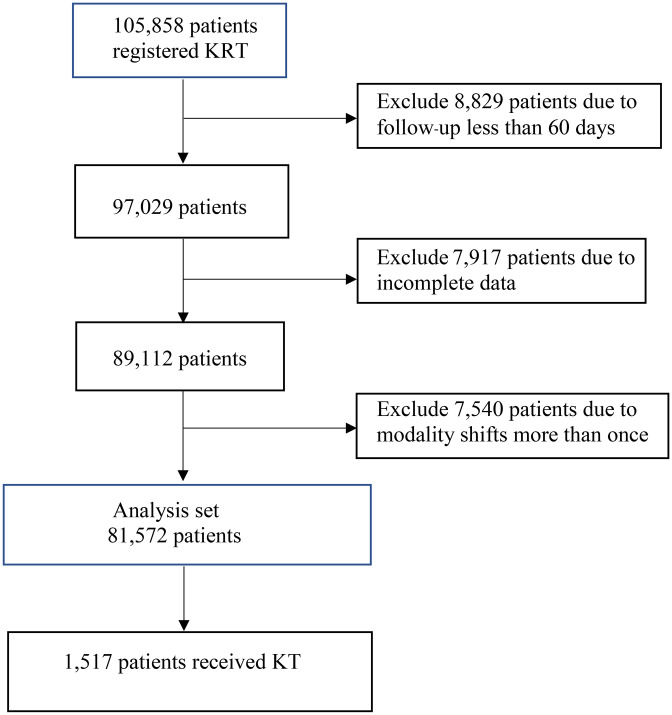
Flow diagram of study patients. A total of 81,572 patients were included in the final analysis, with 1,517 patients receiving KT during the study period. KRT: Kidney Replacement Therapy, KT: Kidney transplantation.

A total of 105,858 patients commenced KRT between January 2013 and December 2021. Fig 1 shows a study flow diagram of patients enrolled in the analysis. Finally, 81,572 patients were included in the analysis. The distribution of dialysis modalities was as follows: 31,756 (38.9%) patients remained on PD; 28,777 (35.3%) on HD; 8,323 (10.2%) transitioned from PD to HD; and 12,716 (15.6%) transitioned from HD to PD. The overall number of patients initially started with PD was 40,079 (49.1%) cases, and 8,323 (20.8%) cases later transitioned to HD. Analogously, 41,493 (50.9%) patients initially started with HD, and 12,716 (30.6%) cases later transitioned to PD. A total of 1,517 (1.9%) patients underwent KT.

Table 1 presents the characteristics of ESKD patients by dialysis modality. The median age at dialysis initiation was highest in patients on HD, followed by those on PD, HD-to-PD transition, and PD-to-HD transition. Hypertension, diabetes, cardiovascular disease, heart failure, and cerebrovascular disease were the predominant comorbidities. Diabetes prevalence was highest in patients on PD, followed by those with HD-to-PD transition, HD, and PD-to-HD transition.

**Table 1:** Patients characteristics classified by dialysis modalities.

Characteristic	Overall (81,572)	PD (31,756)	HD (28,777)	PD-HD (8,323)	HD-PD (12,716)	P-value
Age at start dialysis^a^						<0.001
Median (IQR)	59 (50, 67)	59 (51, 66)	62 (52, 69)	56 (46, 63)	58 (49, 65)	
Age < 60	42,417 (52.0)	16,831 (53.0)	11,511 (40.0)	5,410 (65.0)	7,375 (58.0)	
Age ≥ 60	39,155 (48.0)	14,925 (47.0)	17,266 (60.0)	2,913 (35.0)	5,341 (42.0)	
Sex^b^						<0.001
Male	39,568 (48.5)	15,113 (47.6)	13,734 (47.7)	4,234 (50.9)	6,487 (51.0)	
Female	42,004 (51.5)	16,643 (52.4)	15,043 (52.3)	4,089 (49.1)	6,229 (49.0)	
Diabetes^b^	51,285 (62.9)	20,991 (66.1)	17,389 (60.4)	4,935 (59.3)	7,970 (62.7)	<0.001
Hypertension^b^	76,249 (93.5)	29,889 (94.1)	26,246 (91.2)	7,920 (95.2)	12,194 (95.9)	<0.001
Heart failure^b^	24,061 (29.5)	8,819 (27.8)	8,698 (30.2)	2,450 (29.4)	4,094 (32.2)	<0.001
Cardiovascular disease^b^	30,255 (37.1)	9,943 (31.3)	12,575 (43.7)	3,403 (40.9)	4,334 (34.1)	<0.001
Cerebrovascular disease^b^	15,078 (18.5)	6,070 (19.2)	5,078 (17.6)	1,425 (17.1)	2,505 (19.7)	<0.001
Airway disease^b^	7,597 (9.3)	2,832 (8.9)	2,711 (9.4)	856 (10.0)	1,198 (9.4)	0.001
Liver disease^b^	11,745 (14.4)	3,781 (12.0)	4,516 (15.7)	1,651 (19.8)	1,797 (14.1)	<0.001
HIV^b^	1,010 (1.2)	654 (2.1)	198 (0.7)	72 (0.9)	86 (0.7)	<0.001
Psychiatric problem^b^	4,701 (5.8)	1,663 (5.2)	1,739 (6.0)	527 (6.3)	772 (6.1)	<0.001
Malignancy^b^	3,680 (4.5)	920 (2.9)	1,999 (6.9)	404 (4.9)	357 (2.8)	<0.001
Obesity^b^	511 (0.6)	66 (0.2)	369 (1.3)	37 (0.4)	39 (0.3)	<0.001
Median follow-up time^c^	2.0 (0.9,3.4)	1.9 (0.9, 3.3)	2.2 (0.9, 4.1)	4.3 (2.1, 6.9)	1.8 (0.9, 3.2)	<0.001
Median survival^c^	4.3 (4.3-4.4)	4.2 (4.2 - 4.3)	5.1 (4.9 - 5.3)	8.8 (8.44 - 9.07)	4.9 (4.7 −5.1)	<0.001
Mortality rate^b^	27,500 (33.7)	11,517 (36.3)	9,516 (33.1)	2,685 (32.3)	3,782 (29.7)	<0.001
Kidney transplantation^b^	1,517 (1.9)	1,068 (3.4)	301 (1.0)	141 (1.7)	7 (0.1)	<0.001

^a^years, ^b^ Number (%), ^c^ years (95% Confidence interval).

IQR: interquartile range; PD: peritoneal dialysis; HD: hemodialysis; PD-HD: start with peritoneal dialysis then shift to hemodialysis; HD-PD: start with hemodialysis then shift to peritoneal dialysis.

### Patient survival and associated factors in different dialysis modalities

**Fig 2:**
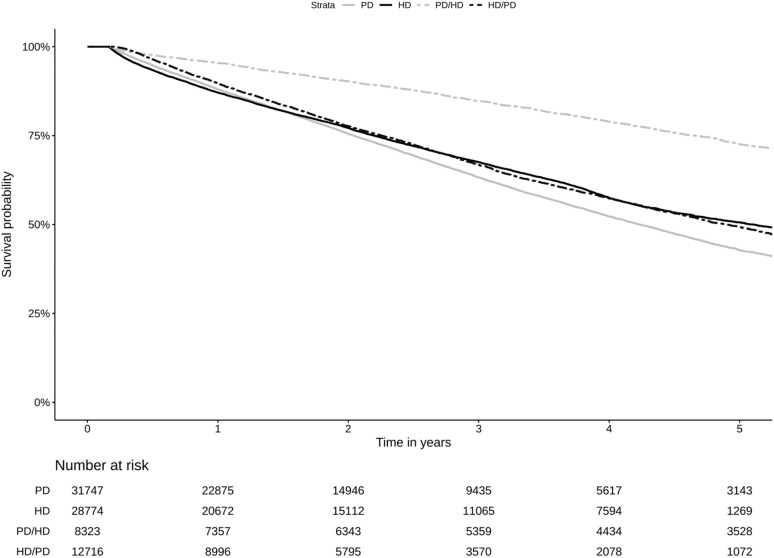
Kaplan–Meier curves comparing patient survival by dialysis modality and transition. Kaplan–Meier curves showing survival among patients who underwent PD, HD, started with PD and later transitioned to HD, or started with HD and later transitioned to PD. The survival difference among the groups was statistically significant (log-rank test, *p* < 0.001). PD: peritoneal dialysis; HD: hemodialysis; PD-HD: start with peritoneal dialysis then shift to hemodialysis; HD-PD: start with hemodialysis then shift to peritoneal dialysis.

Fig 2 shows patient survival rates among different dialysis modalities. The 1, 3 and 5-year patient survival rates in the PD-to-HD transition were 95.4, 84.7, and 72.6%, respectively, the highest among all groups. The 1-year survival rates for PD, HD, and HD-to-PD transition were 88.0, 87.1, and 89.7%, respectively, with minimal differences. The 3- and 5-year patient survival rates were lowest in patients who stayed on PD only (Fig 2 and S1 Table). The median (IQR) follow-up time for the PD-to-HD transition was 4.3 (2.1, 6.9) years, longer than that for PD, HD, and HD-to-PD transition (p < 0.001) (Table 1). The median (IQR) follow-up time for patients who stayed on PD only was 1.9 (0.9, 3.3) years, compared to 2.2 (0.9, 4.1) years for those on HD (p 0.32) and 1.8 (0.9, 3.2) years for those in the HD-to-PD transition (p 0.78). Among dialysis modalities, the overall mortality rate was highest in patients who remained on PD (p < 0.001) (Table 1 and S1 Table).

**Fig 3:**
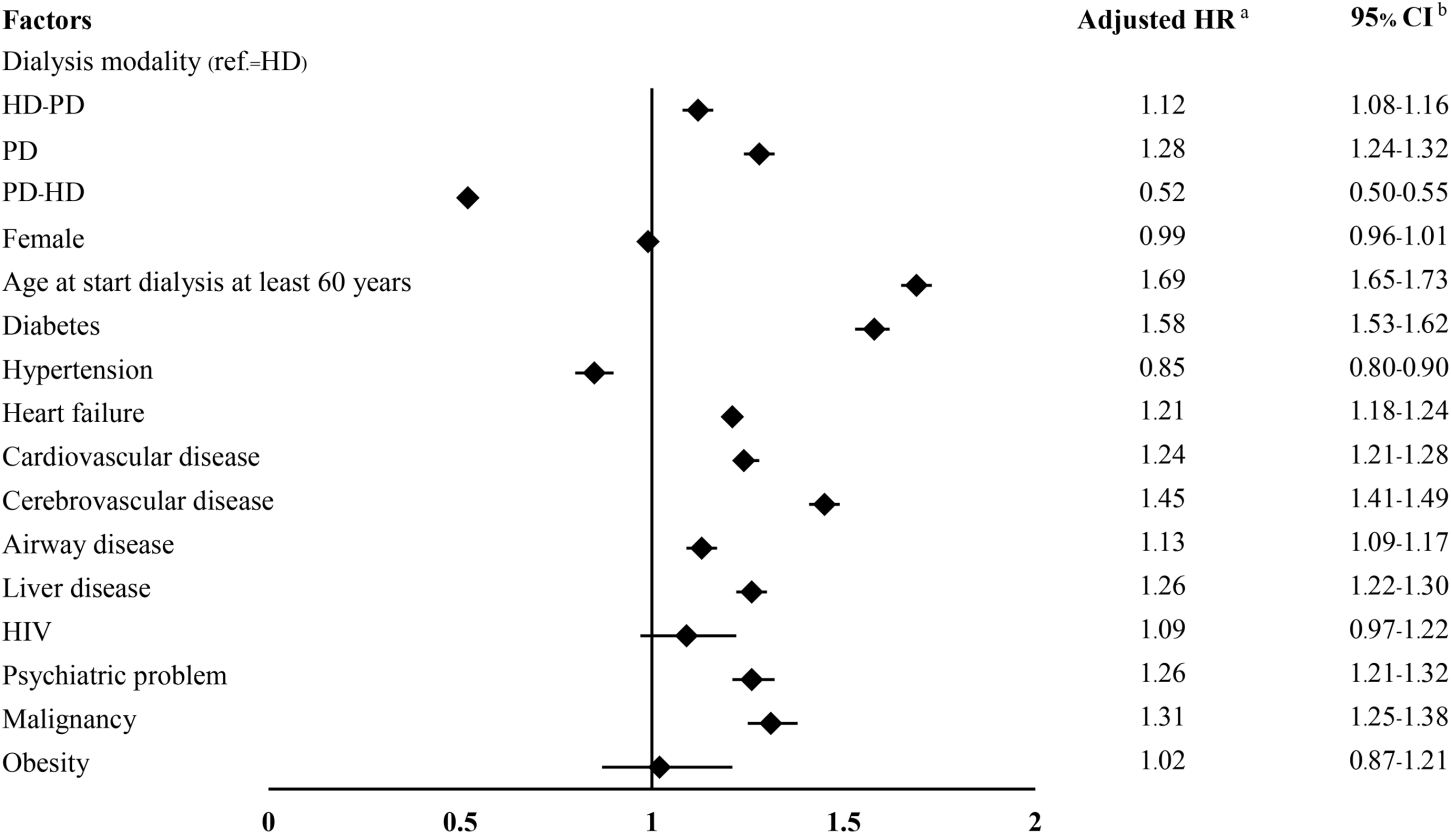
Adjusted hazard ratios for all-cause mortality by dialysis modality and clinical predictors. Forest plot showing adjusted HRs with 95% confidence intervals for predictors of mortality. Significant predictors included dialysis modality, age 60 years or older at dialysis initiation, diabetes, heart failure, cardiovascular disease, cerebrovascular disease, airway disease, liver disease, psychiatric disorders, and malignancy. PD: peritoneal dialysis; HD: hemodialysis; PD-HD: start with peritoneal dialysis then shift to hemodialysis; HD-PD: start with hemodialysis then shift to peritoneal dialysis. ^a^ Hazard ratio; ^b^ confidence interval. HRs were estimated from a multivariable Cox model including dialysis modality and patient characteristics, adjusted for age, sex, and comorbidities.

Fig 3 shows the multivariable Cox proportional hazards model. In addition to dialysis modality, several patient-level clinical factors were independently associated with higher mortality. These included age at dialysis initiation 60 years or older, diabetes, heart failure, cardiovascular disease, cerebrovascular disease, airway disease, liver disease, psychiatric disorders, and malignancy. Hypertension, HIV, and obesity did not increase mortality risk.

Table 2 illustrates the association between dialysis modality and mortality obtained from subgroup analyses designed to evaluate effect modification by key patient characteristics. For the purpose of these subgroup analyses, a combined comorbidity variable was employed as a binary indicator, differing from the individual adjustment of comorbidities presented in the main model (Fig 3). Across various subgroups, including patients aged less than 60 years, those with or without diabetes, and individuals characterized by the presence of combined comorbidities, the PD-to-HD transition consistently demonstrated the lowest risk of mortality compared to HD-only. Furthermore, PD-only consistently showed a higher risk of mortality compared to HD-only across all subgroups examined.

**Table 2:** Adjusted hazard ratios for all-cause mortality associated with dialysis modality, stratified by patient subgroups.

Characteristics	Age < 60			Age ≥ 60		
	Adjusted HR^a^	95% CI^b^	P-value	Adjusted HR^a^	95% CI^b^	P-value
HD	Ref			1.76	1.68-1.84	<0.001
HD-PD	1.16	1.09-1.23	<0.001	1.92	1.81-2.03	<0.001
PD	1.35	1.29-1.42	<0.001	2.16	2.07-2.26	<0.001
PD-HD	0.51	0.47-0.54	<0.001	0.97	0.91-1.04	0.764
	**No Diabetes**			**Diabetes**		
HD	Ref			1.44	1.38-1.51	<0.001
HD-PD	0.99	0.92-1.06	0.8	1.68	1.60-1.78	<0.001
PD	1.19	1.13-1.25	<0.001	1.89	1.81-1.97	<0.001
PD-HD	0.46	0.42-0.50	<0.001	0.80	0.75-0.85	<0.001
	**No combined comorbidities**			**Combined comorbidities**		
HD	Ref			1.63	1.52-1.74	<0.001
HD-PD	0.99	0.95-1.03	0.7	1.97	1.72-2.26	<0.001
PD	1.19	1.16-1.22	<0.001	2.06	1.89-2.24	<0.001
PD-HD	0.46	0.44-0.48	<0.001	1.01	0.86-1.18	1.00

* Combined comorbidities (Age ≥ 60 years, diabetes, cardiovascular disease and cerebrovascular disease).

^a^Hazard Ratio, ^b^95% Confidence Interval.

PD: peritoneal dialysis; HD: hemodialysis; PD-HD: start with peritoneal dialysis then shift to hemodialysis; HD-PD: start with hemodialysis then shift to peritoneal dialysis.

Age: adjusted HR for diabetes mellitus, gender, and all other individual comorbid conditions listed.

Diabetes: adjusted HR for age, gender, and all other individual comorbid conditions listed.

Combined comorbidity: adjusted HR for age gender, diabetes, and other individual comorbidities except cardiovascular disease and cerebrovascular disease.

### Characteristics of kidney transplantation in different dialysis modalities

The number of dialysis patients who received KT was 1,517 out of 81,572 (1.9%) cases. Table 3 shows the characteristics of dialysis patients who received KT. The majority of dialysis modalities in KT recipients were PD (70.4%), followed by HD (19.8%) and dialysis transition (9.8%). The median (IQR) age of KT recipients was 35.7 (23.8, 47.1) years which was less than 50 years. The main type of KT was from deceased donor (71.7%). The percentage of PD patients receiving a living related donor was higher than that for HD patients, followed by those who underwent dialysis transition. The overall median (IQR) waiting time to KT in was 94.5 (69, 129.3) months. The waiting time was longest in patients with dialysis transition (136.8 [125.2, 151.0] months), followed by those on PD (101.2 [73.1, 131.1] months), and those on HD (70.0 [58.8, 82.5] months). Most KT recipients had any HLA mismatches which were HLA-A, HLA-B or HLA-DR mismatches. Patients developed antibody-mediated rejection were 14.8%, 8.9% developed acute cellular rejection, and 22.5% developed delayed graft function.

**Table 3:** Characteristics of patients received kidney transplantation.

Characters	HD	PD	PD and HD	Overall	P-value
	(301)	(1,068)	(148)	(1,517)	
Age at KT^a^	43.1 (31.0, 52.2)	33.5 (21.3, 44.6)	37.3 (26.7, 48.0)	35.7 (23.8, 47.1)	< 0.001
Sex					0.545
Male^b^	175 (58.1)	644 (60.3)	94 (63.5)	913 (60.2)	
Female^b^	126 (41.9)	424 (39.7)	54 (36.5)	604 (39.8)	
Diabetes^b^	14 (4.7)	111 (10.4)	6 (4.0)	131 (8.6)	< 0.001
Hypertension^b^	238 (79.1)	496 (46.4)	137 (92.6)	871 (57.4)	< 0.001
Cardiac disease^b^	143 (47.5)	259 (24.3)	82 (55.4)	484 (31.9)	< 0.001
Non-cardiac disease^b^	137 (45.5)	272 (25.5)	70 (47.3)	479 (31.6)	< 0.001
Waiting time to KT^c^	70.0 (58.8, 82.5)	101.2 (73.1, 131.1)	136.8 (125.2, 151)	94.5 (69, 129.3)	< 0.001
Types of donors^b^					< 0.001
Living	80 (26.6)	327 (30.6)	23 (15.5)	430 (28.3)	
Deceased	221 (73.4)	741 (69.4)	125 (84.5)	1087 (71.7)	
HLA mismatch^b^					
Any HLA mismatch	107 (35.5)	428 (40.1)	72 (48.6)	607 (40.0)	0.022
One HLA mismatch	19 (6.3)	68 (6.4)	10 (6.8)	97(6.4)	0.783
Two HLA mismatch	88 (29.2)	360 (33.7)	62 (41.9)	510 (33.6)	0.028
Three HLA mismatch	76 (25.2)	301 (28.2)	53 (35.8)	430 (28.3)	0.064
HLA-A mismatch	92 (30.6)	355 (33.2)	60 (40.5)	507 (33.4)	0.134
HLA-B mismatch	89 (29.6)	374 (35.0)	64 (43.2)	527 (34.7)	0.011
HLA-DR mismatch	90 (29.9)	360 (33.7)	63 (42.6)	513 (33.8)	0.041
Antibody-mediated rejection^b^	44 (14.6)	165 (15.4)	16 (10.8)	225 (14.8)	0.017
Acute cellular rejection^b^	19 (6.3)	108 (10.1)	9 (6.1)	136 (8.9)	0.192
Delay graft function^b^	58 (19.3)	237 (22.2)	46 (31.1)	341 (22.5)	0.007
					

^a^years, median (IQR), ^b^N (%),^c^months, median (IQR).

HD: hemodialysis; PD: peritoneal dialysis; KT: kidney transplantation.

### Patient survival and associated factors after KT in different dialysis modalities

**Fig 4:**
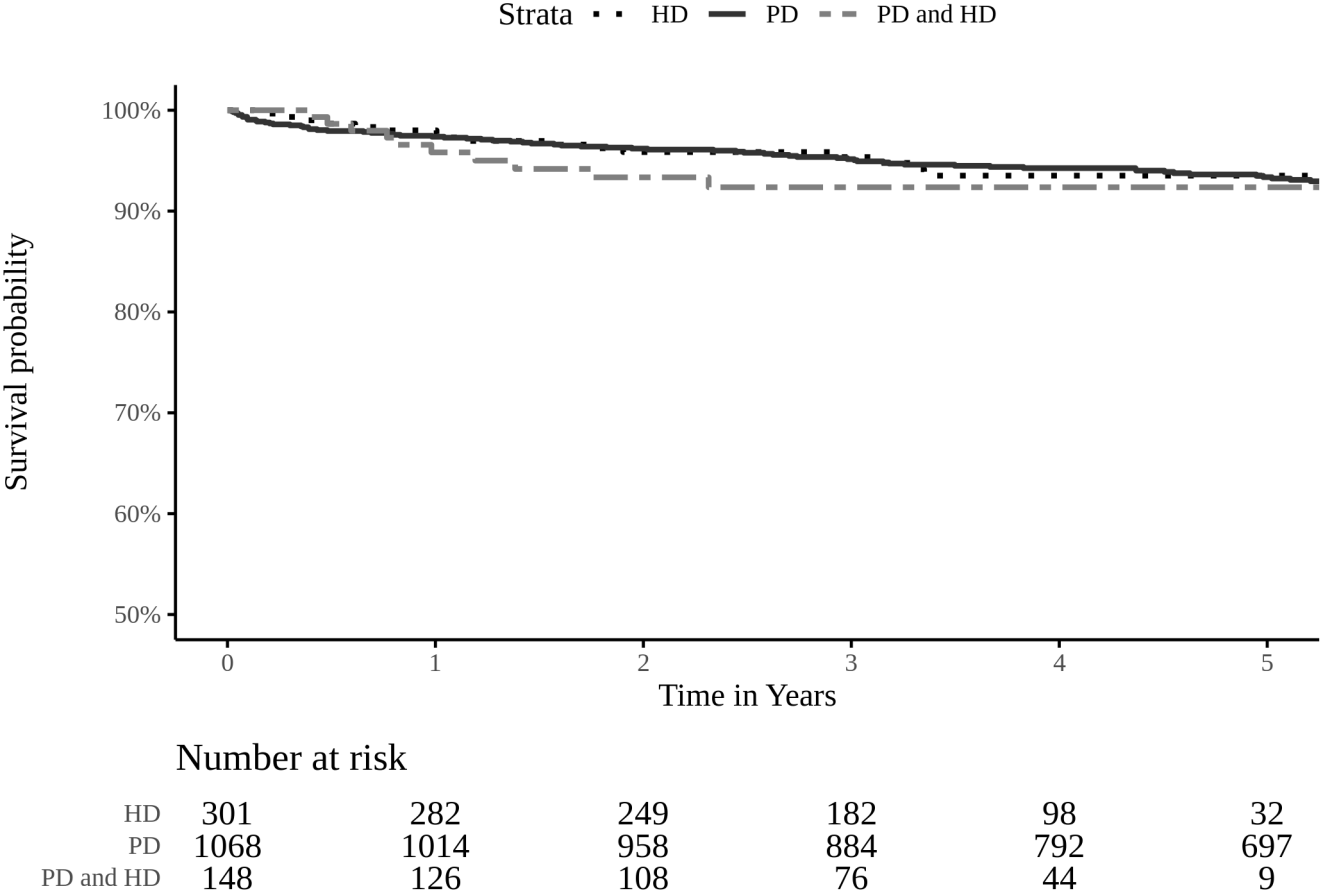
Patient survival after receiving kidney transplantation classified by dialysis modalities. Kaplan–Meier curves showing post-transplant survival among patients who underwent PD, HD, or changed dialysis modality between PD and HD prior to transplantation. The survival difference among the groups was not statistically significant (log-rank test, *p* = 0.06). PD: peritoneal dialysis; HD: hemodialysis; PD and HD: patient with a history of changing dialysis modality between peritoneal dialysis and hemodialysis.

**Fig 5:**
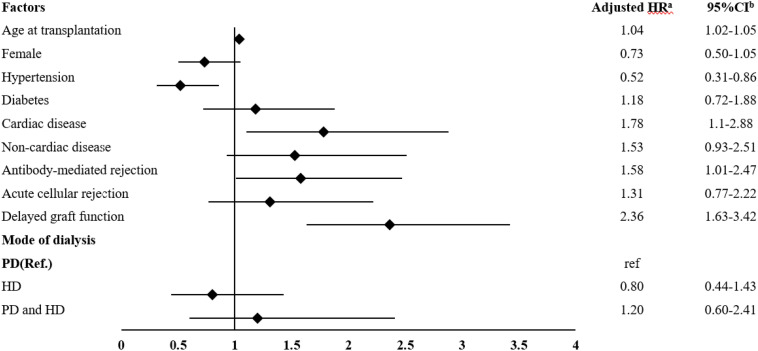
Factors associated with mortality in patients receiving kidney transplantation. Forest plot showing adjusted HRs with 95% confidence intervals for predictors of mortality among kidney transplant recipients. Factors significantly associated with increased mortality included age at transplantation, cardiovascular disease, antibody-mediated rejection, and delayed graft function. Dialysis modality prior to transplantation was not associated with mortality. PD: peritoneal dialysis; HD: hemodialysis; PD and HD: patient with a history of changing dialysis modality between peritoneal dialysis and hemodialysis. ^a^ Hazard ratio; ^b^ confidence interval.

The median (IQR) follow-up time in KT recipients who started with PD was 75.8 (46.8, 107.1) months which was longer than those who started with HD, and those who underwent dialysis transition. The overall mortality in these patients was 8.5%. The mortality rate was highest in the PD group. To accurately assess the impact of prior dialysis modality on post-transplant survival, it is crucial to account for differences in follow-up time. Kaplan-Meier survival analysis and a multivariable Cox proportional hazards model were employed to appropriately handle censored data and varying follow-up times, providing time-adjusted survival estimates. The 1, 2, 3 and 5-year survival rates in all groups were above 90%, which were comparable among the three groups (Fig 4 and S2 Table). Factors significantly associated with mortality among KT recipients included age at the time of KT, cardiac disease, antibody-mediated rejection, and delayed graft function. Dialysis modality was not a risk factor for mortality in these patients (Fig 5).

## Discussion

The findings of this study provide significant insights into patient survival across various dialysis modalities under Thailand’s PD-first policy within the UHC system, as well as post-KT survival. Patients who initiated PD and subsequently transitioned to HD had better survival rates than those who remained on PD, HD, or transitioned from HD to PD. Independent factors associated with higher mortality across all modalities included advanced age at dialysis initiation, diabetes, and comorbidites such as cardiovascular disease, heart failure, cerebrovascular disease, airway disease, mental health disorders, and malignancies. Among patients with these factors, those who started dialysis with PD and transitioned to HD demonstrated the highest survival rates. Patients who had previously been on PD were more likely to undergo KT. Post-KT survival rates were comparable among patients on PD, HD, or transitioning between PD and HD. Factors associated with mortality after KT included age at KT, cardiac disease, antibody-mediated rejection, and delayed graft function.

Several publications report similar survival and mortality rates between PD and HD in patients with comparable characteristics [5–7,14–16]. Analysis of the Netherlands Cooperative Study on the Adequacy of Dialysis 2 revealed no statistically significant difference in adjusted mortality rates between HD and PD during the first two years of dialysis [17]. Long-term use of PD, especially among patients aged 60 years or older, is associated with an increased mortality rate [17]. Some studies have examined survival rates in patients who remained on PD or HD compared to those who transitioned between these modalities. A matched case-control study from a single center showed higher 1-, 3-, and 5-year survival rates in patients who stayed on PD than in those who started on HD and later transitioned to PD, though cumulative survival did not differ significantly [18]. A systematic review and meta-analysis demonstrated that patients transition from HD to PD had lower survival compared to those who remained on PD [19]. A study from Taiwan revealed that patients transitioning from PD to HD had a higher risk of death compared to those on HD alone [20]. A multinational study using ANZDATA, CORR, ERA Registry, and the USRDS databases found high mortality rates after transitioning from PD to HD, particularly in the early post-transition period, among elderly patients, and in those with prolonged PD vintage [21].

The lower mortality among patients transitioning from PD to HD in our findings is a striking observation and provides support for the effectiveness of an integrative care approach within the specific context of the Thai healthcare system**.** This result aligns with a previous single-center study [22], suggesting it may be a consistent pattern in our setting. PD and HD should be viewed as complementary rather than competing modalities. The current study shows that the Thai PD-first policy is more cost-effective than an HD-first approach under the current willingness-to-pay threshold [23]. The Thai PD-first policy permitted reimbursement for initiating HD in patients with contraindications to PD, transitioning from failed PD to HD, and vice versa. A higher proportion of KT recipients in our study had previously received PD, and among them, living-related donation was more common compared to HD patients. While this analysis did not formally compare the likelihood of undergoing KT across dialysis modalities, this distribution may reflect contextual factors including historical access constraints, patient selection, and policy influences under the PD-first approach. In Thailand, living kidney donation is legally permitted only from first-degree relatives or legally recognized spouses. As PD is a home-based therapy and there is no national assisted PD program, family members are often involved in the patient’s daily care. This close caregiving relationship may increase awareness of disease burden and foster a greater willingness to donate. In addition, the scarcity of deceased donor organs in Thailand may lead families to consider living donation more often, particularly when the patient is already receiving PD at home. Although our dataset does not allow for causal inference, these contextual factors may partly explain the higher proportion of living-related KT among PD patients. Further qualitative or prospective research is warranted to investigate this hypothesis.

These findings should be interpreted within the broader policy context. While the present study did not assess national trends in KRT incidence or coverage, previous studies have associated the PD-first policy with expanded dialysis access and improved cost containment under Thailand’s UHC system. Our results provide additional insights into how modality choice and patient outcomes may reflect both clinical needs and the structural features of national policy frameworks.

Previous studies show that residual kidney function (RKF) is preserved longer in patients on PD than in those on HD [24–26]. Initiating PD in patients with ESKD allows them to postpone vascular access creation for future HD, thereby preserving vascular integrity and reducing the risk of access-related complications [27–29]. The benefits of PD diminish over time due to progressive loss of RKF and declining peritoneal membrane function, leading to inadequate clearance and ultrafiltration [30]. PD patients who transition to HD may do so due to complications such as fluid overload, or PD-related infection, or social constraints related to PD prescriptions [31–33]. The superior survival observed in our PD-to-HD cohort, which contrasts with literature where PD failure typically implies worse outcomes, can be largely attributed to the highly flexible and patient-centered integrative care approach within the Thai healthcare system. Unlike more rigid systems, Thailand’s policy allows for ready transition between dialysis modalities based on evolving patient needs. This means a proactive approach to switching patients from PD to HD when early signs of PD inadequacy or complications arise, or when social circumstances impede effective PD, rather than waiting for severe, irreversible complications. Consequently, patients in our PD-to-HD group often represent those who were successfully managed by transitioning to the most appropriate modality at the right time, thereby avoiding the severe adverse outcomes typically associated with late PD failure. However, it is crucial to interpret this observed superior survival with caution, as it potentially reflects the presence of unmeasured confounding and survivor bias. While the observed superior survival in our PD-to-HD cohort is notable and aligns with the concept of an integrative care approach, it must be interpreted with caution. Our previous attributing of this improved survival to more favorable baseline characteristics (e.g., younger age, lower prevalence of diabetes) and the benefits of preserved RKF, while partially valid, may not fully capture the underlying reasons, especially after multivariable adjustments. Those who survive long enough to transition may represent a healthier subgroup with a better overall prognosis– a phenomenon consistent with survivor bias. This suggests that only patients with sufficient clinical stability and fewer complications on PD are able to successfully make such a transition. Therefore, the persistence of superior survival in this group, even after adjustment for measured confounders, suggests that residual unmeasured confounding also plays a significant role, limiting the direct causal inference that the transition itself is solely responsible for the improved survival.

HD may offer better volume control and metabolic correction in certain clinical scenarios. For patients transitioning from HD to PD, medical factors such as poor cardiac conditions, hemodynamic instability, or vascular access problems often play a role [34,35]. Non-medical factors, including physician and provider preferences, may also limit the transition from HD to PD [36]. A prior study indicated that prolonged time on HD before transitioning to PD was associated with increased mortality risk [37].

Our study lacks data on the reasons for PD-to-HD or HD-to-PD transitions, making it difficult to determine whether the survival benefit is due to the transition itself or due to patient selection and residual confounding. We support timely transition from PD to HD when dialysis adequacy cannot be achieved, ultrafiltration failure occurs, or social factors necessitate the switch, as unplanned transitions are associated with higher morbidity and mortality risks [38]. Similarly, transitioning from HD to PD should be carefully planned to ensure proper adaptation and alignment with individual patient circumstances [39]. Future studies incorporating clinical indications for switching between dialysis modalities are needed to clarify these findings.

The reasons for patients remaining on PD may be due to advanced age, a high comorbidity burden, or frailty, which make them unsuitable for HD [40,41]. Our results identified several clinical factors that were independently associated with higher mortality across dialysis modalities, including advanced age, diabetes, heart failure, cardiovascular disease, cerebrovascular disease, airway disease, liver disease, psychiatric disorders, and malignancies. While these findings help characterize patients at higher risk of death, they do not directly explain which factors are associated with remaining on PD. Hypertension appeared to be associated with a lower risk of mortality in our analysis. This finding is likely attributable to chance or residual confounding rather than a true protective effect. Previous studies have also reported similar paradoxical associations, which may result from factors such as better medical surveillance or misclassification [42,43]. Therefore, this result should be interpreted with caution and warrants further investigation.

Socioeconomic barriers may also contribute to the inability of some patients to transition from PD to HD when clinically indicated [44]. This study reflects real-world practice under Thailand’s PD First policy, wherein dialysis modality is often influenced by national policy and patients’ financial capacity, rather than solely by clinical criteria. The absence of a national assisted PD program requires that patients on PD rely heavily on family or caregiver support. In contrast, patients opting for HD often incur out-of-pocket expenses, which may reflect higher socioeconomic status. Previous reports have shown that HD patients are more likely to pay additional costs for treatment compared to PD patients [45]. These differences in socioeconomic status, caregiver availability, and treatment access would likely underlie the observed variation in survival across dialysis modalities, particularly the lower survival among patients who remained on PD throughout the study period. Our findings thus indirectly support the notion that socioeconomic factors may influence treatment choice and, consequently, observed survival patterns in this population. To address these challenges, the NHSO should consider strategies to improve PD outcomes for patients unable to transition to HD. Although automated peritoneal dialysis (APD) is covered under UHC, it may not be suitable for all patients [46]. Assisted PD or non-glucose-based PD solutions may help support patients facing obstacles in transitioning to HD due to medical or non-medical factors. Individualized care goals focusing on quality of life, rather than simply extending lifespan, should be prioritized for frail patients or those with high comorbidity burdens and integrated as key performance indicators within the healthcare system [47]. Such approaches may position PD as a form of comfort care, focusing on symptom management, including volume management [48].

Although the associations between age, comorbidities, and mortality are well known, our findings confirm their significance in a policy-driven dialysis system with limited modality choice. Similar predictors—age over 65 years, diabetes, high comorbidity burden, and malnutrition—were also reported in a Colombian cohort [49], supporting the broader applicability of these risk factors across health systems.

Patients on dialysis should be proactively informed about KT options and referred for evaluation. Our study reported favorable overall survival rates for KT recipients, with five-year survival rates of 93.1%. Post-KT patient survival was comparable between PD and HD consistent with findings from systematic reviews and meta-analyses [50]. Our analysis indicated that survival after kidney transplantation was not affected by dialysis modalities despite variation in KT waiting time and comorbidities prior KT. It would suggest that policies regarding dialysis modality should be maximally flexible for patients who are KT candidates, to maximize their chances of getting to KT, since their survival afterward appears to be equivalent. However, due to specific limitations in our available dataset, we were unfortunately unable to calculate the precise likelihood or incidence of KT by dialysis modalities. Therefore, our approach was limited to describing the baseline characteristics of patients who ultimately underwent KT (as presented in Table 3) and analyzing how their prior dialysis modality was associated with post-transplant survival. While this does not directly address the likelihood of receiving a transplant, it provides crucial insights into outcomes among those who did receive one, differentiating between prior HD and PD exposure. Notably, patients on PD had higher likelihood of receiving KT compared to those on HD, likely due to their younger age and fewer non-medical barriers. Similar to other studies, age, comorbidities, acute rejection, and delayed graft function were the primary risk factors for mortality after KT, rather than dialysis modality [51].

While this study provides valuable insights, it is important to acknowledge its limitations. First, more than 8% of patients were excluded due to incomplete or inconsistent data. The missing information primarily involved: (1) absence of documented comorbidities, (2) patients with only a single dialysis record followed by immediate loss to follow-up, and (3) inconsistencies such as death dates recorded prior to dialysis initiation. Based on an intuitive review of data patterns, we assume the missingness to be missing completely at random (MCAR); however, no formal statistical test was conducted to confirm this assumption. As such, the potential impact of missing data on the generalizability of findings cannot be fully ruled out. Second, there is a lack of data on the reasons for transitioning between modalities and the reasons for remaining on a specific modality despite having conditions that warrant transition. Without accounting for these factors, the observed differences in patient survival between dialysis modalities and transitions may be influenced by unmeasured confounders. Third, we were unable to adjust for potential confounders such as socioeconomic status, nutritional status, frailty status, and early dropout from dialysis due to data unavailability. These factors may influence survival and transplantation outcomes. For example, low socioeconomic status can be barrier to transitioning to HD and KT, while nutritional status is a known predictor of mortality in dialysis patients. Fourth, the timing of comorbidity development, such as cardiovascular disease, could not be ascertained. These comorbidities may have predated dialysis initiation, arisen as complications of dialysis, or resulted from disease progression. Fifth, the causes of death were not identified, limiting the ability to propose specific quality improvement strategies. Sixth, dialysis modalities were not randomly assigned, limiting causal inferences. Lastly, the inability to distinguish between patients on HD due to medical contraindications to PD and those who opted for HD by self-funding, as such detailed financial or clinical information was not available in the administrative database. This limitation may affect the interpretation of modality choice patterns under the PD-first policy. Future studies with more comprehensive data, including socioeconomic and clinical factors influencing dialysis modality selection, will be needed to address these limitations. Despite these limitations, the strengths of this study include a large, nationally representative dataset encompassing most ESKD patients on dialysis in Thailand and an extended follow-up period. The inclusion of modality transitions provides valuable real-world insights into ESKD care practices.

This is the first study to demonstrate patient survival across different dialysis modalities, including KT, under Thailand’s PD-first policy within the UHC framework. The findings provide valuable insights for policymakers in designing cost-effective and equitable KRT strategies. However, requiring patients without medical contraindications to PD to self-fund HD has raised ethical concerns regarding patient autonomy and financial burden. In response, Thailand’s national policy is undergoing a transition from a strict PD-first approach to a shared decision-making (SDM) model. This shift aims to promote greater patient involvement in dialysis modality selection while preserving the sustainability of the UHC system. The evolving policy landscape should be considered when interpreting modality trends and planning future dialysis service delivery. Under this new SDM model, patients are now enabled to choose their initial dialysis modality (either PD or HD) based on comprehensive clinical assessment, patient preference, and informed consent. Importantly, for patients who choose or are clinically indicated for HD as their initial modality, the costs for HD sessions are fully reimbursed by the UHC scheme, similar to PD. This addresses the previous ethical concerns regarding financial barriers to HD access.

The long-term financial sustainability of dialysis care under UHC, particularly with the potential for increased HD uptake under SDM, is a significant and ongoing challenge. Measures in place or under consideration to address this include prioritizing kidney transplantation, continuing to promote cost-effective home-based therapies like PD, conducting regular cost-effectiveness analyses, enhancing early detection and prevention of CKD progression, and optimizing resource allocation. This evolving policy environment, while aiming to improve patient-centered care, presents a critical area for future policy evaluation and management within the NHSO to ensure the continued sustainability of UHC benefits for kidney replacement therapy. As the shift toward SDM has been associated with increasing HD uptake and a decline in PD initiation, both HD and PD should be viewed as complementary options. Integrating both modalities within a patient-centered framework may enhance overall care quality and outcomes. Future research should address current data limitations by incorporating additional variables such as socioeconomic status, frailty indices, and cause-specific mortality. These data would enable more accurate assessment of dialysis outcomes and support personalized modality selection for patients with ESKD.

## Conclusions

This study offers valuable insights into the survival outcomes associated with different dialysis modalities under Thailand’s PD-first policy within UHC system. The findings highlight the advantages of a strategic treatment pathway that begins with PD, followed by a planned transition to HD or KT. This stepwise approach optimizes healthcare resource allocation, reduces the risk of long-term complications, and ultimately improves patient outcomes.

## Supporting information

S1 Table Total follow-up times, mortality rates, and survival rates classified by dialysis modalities. (DOCX)

S2 Table Mortality and survival rates comparing among groups of dialysis patients who received kidney transplantation. (DOCX)
