# Supplementary material for: Patient survival and kidney transplantation in different dialysis modalities under PD First Policy Thailand
Source: PLoS One. 2025 Nov 19;20(11):e0336954. doi: 10.1371/journal.pone.0336954 (PMC12629467; doi:10.1371/journal.pone.0336954)
Supplement: S1 Table — (DOCX) [file pone.0336954.s001.docx]

**Supplementary Table 1 Mortality and** s**urvival rates classified by dialysis modalities**

|  | **PD** | **HD** | **PD-HD** | **HD-PD** | **P-value** |
| --- | --- | --- | --- | --- | --- |
| Total number of patients^a^ | 31,756 (38.9) | 28,774 (35.3) | 8,323 (10.2) | 12,716 (15.6) | <0.001 |
| Total follow up time  (Person-Year) | 74,788.5 | 69,973.1 | 38,356.5 | 28,872.1 |  |
| Mortality rates  (per 100 Person-Years) | 15.4 | 13.6 | 7.0 | 13.1 | <0.001 |
| Kaplan-Meier survival analysis |  |  |  |  |  |
| 1-year survival rates^b^ | 88.0  (87.7 – 88.4) | 87.1  (86.7 – 87.5) | 95.4  (95.0 – 95.9) | 89.7  (89.1 – 90.3) | <0.001 |
| 3-year survival rates^b^ | 63.2  (62.6 – 63.9) | 67.5  (66.9 – 68.2) | 84.7  (83.9 – 85.5) | 66.9  (65.8 – 67.9) | <0.001 |
| 5-year survival rates^b^ | 42.8  (41.9 – 43.6) | 50.6  (49.6 – 51.6) | 72.6  (71.5 – 73.7) | 49.4  (47.9 – 50.8) | <0.001 |

^a^ Number (%), ^b^ % (95% Confidence interval)

PD: peritoneal dialysis; HD: hemodialysis; PD-HD: start with peritoneal dialysis then shift to hemodialysis; HD-PD: start with hemodialysis then shift to peritoneal dialysis
