# Supplementary material for: Patient survival and kidney transplantation in different dialysis modalities under PD First Policy Thailand
Source: PLoS One. 2025 Nov 19;20(11):e0336954. doi: 10.1371/journal.pone.0336954 (PMC12629467; doi:10.1371/journal.pone.0336954)
Supplement: S2 Table — (DOCX) [file pone.0336954.s002.docx]

**Supplementary Table 2 Mortality and survival rates** **comparing among groups of dialysis patients who received kidney transplantation**

|  | **PD**  **(1,068)** | **HD**  **(301)** | **PD and HD**  **(148)** | **Overall**  **(1,517)** | **P-value** |
| --- | --- | --- | --- | --- | --- |
| Total follow-up time after KT^a^ | 75.8  (46.8, 107.1) | 40  (28.4, 51.3) | 37  (22.2, 50.2) | 59  (36.0, 93.3) | <0.001 |
| Mortality^b^ | 103 (9.6) | 16 (5.3) | 10 (6.8) | 129 (8.5) | 0.043 |
| **Patient survival rates**^c^ |  |  |  |  |  |
| 1-year survival rates | 97.4  (96.4-98.3) | 98.0  (96.4-99.6) | 95.8  (92.6-99.2) | 97.4  (96.5–98.2) | 0.439 |
| 2-year survival rates | 96.2  (95.0-97.4) | 95.9  (93.6-98.2) | 93.3  (89.2-97.7) | 95.9  (94.9-96.9) | 0.246 |
| 3-year survival rates | 95.2  (93.8-96.5) | 95.4  (92.9-97.9) | 92.4  (87.9-97.1) | 94.9  (93.8-96.1) | 0.362 |
| 5-year survival rates | 93.4  (91.8-94.9) | 93.5  (90.4-96.8) | 92.4  (87.9-97.1) | 93.1  (91.8-94.5) | 0.922 |

^a^ month, median (IQR), ^b^ Number (%), ^c^ % (95% Confidence interval)

PD: peritoneal dialysis; HD: hemodialysis; KT: kidney transplantation
